# Circadian rhythms of neuronal and astrocytic glutamate transporters in the suprachiasmatic nucleus of mice

**DOI:** 10.3389/fncel.2026.1875844

**Published:** 2026-08-05

**Authors:** Hussein Haidar, Amira A. H. Ali, Charlotte von Gall, Federica Tundo-Lavalle

**Affiliations:** 1 Institute of Anatomy II, Medical Faculty, Heinrich-Heine-University, Düsseldorf, Germany; 2 Department of Human Anatomy and Embryology, Faculty of Medicine, Mansoura University, Mansoura, Egypt

**Keywords:** circadian, diurnal, GLAST, glutamatergic tripartite synapse, suprachiasmatic nucleus, VGLUT1, VGLUT2

## Abstract

Retinal ganglion cells release glutamate at their synapses, transmitting visual signals via the optic nerve to brain regions involved in vision and light-dependent functions. Glutamatergic input to the circadian pacemaker, the suprachiasmatic nucleus (SCN), is essential for photoentrainment of circadian rhythms. Glutamate transporters are crucial for efficient transmission in the tripartite synapse formed by presynaptic terminals, postsynaptic neurons and astrocytes. However, it remains unclear whether this glutamatergic synapse is modulated by rhythmic environmental lighting conditions. In this study, neuronal (VGLUT1 and VGLUT2) and astrocytic (GLAST) glutamate transporters of 8–12 weeks old male C57Bl/6 J mice were analyzed in the SCN of mice exposed to different lighting paradigms: a standard 12:12-h light–dark cycle (LD), constant darkness (DD) and constant light (LL), using immunofluorescence and confocal laser microscopy. Under LD, all three transporters exhibited time-of-day–dependent rhythms: VGLUT1 and GLAST peaked during the light phase, while VGLUT2 peaked during the dark phase. This suggests that VGLUT1 and GLAST are more strongly coupled and that VGLUT2 is a complementary system, so that the strength or efficiency of the light information can be modulated depending on the time of day. Furthermore, the rhythms of neuronal glutamate transporters persisted under DD and LL, indicating robust circadian control. Our study thus supports the hypothesis that the circadian system may regulate its own input.

## 1 Introduction

Circadian rhythms are endogenous oscillations of bodily functions generated by an internal clock that continue to run even in the absence of rhythmic signals from the environment with a period length of approximately 24-h (Johnson et al., 2003; Welsh et al., 1995). At the cellular level, circadian rhythms are driven by a self-sustaining molecular clockwork consisting of interconnected transcriptional/translational feedback loops of clock genes, which drives rhythmic gene expression via E-box elements (Reppert and Weaver, 2002). However, the phase and period length of circadian rhythms can be adjusted by rhythmic light information from the environment, a process called photoentrainment (Ashton et al., 2022; Johnson et al., 1988). In addition, even without an intact internal clock, the activity of nocturnal rodents can be directly inhibited by light, a process called negative masking (Morin et al., 2010). In mammals, circadian rhythms are controlled by a hierarchically organized circadian system, the main function of which is to anticipate rhythmic events in the environment and coordinate various rhythmic processes within the body (Korf and von Gall, 2022). Chronic disturbances of the circadian system, chronodisruption, can lead to health complications, and on the other hand, chronic diseases are often associated with chronodisruption (Fishbein et al., 2021; Lucassen et al., 2016). Artificial light at night (ALAN) can affect the circadian system, mainly by shifting the phase of the internal clock, which can lead to irregular sleep patterns, daytime sleepiness, and cognitive problems in humans (Blume et al., 2019; Korf et al., 2025). Chronic ALAN exposure is associated with metabolic diseases such as obesity and diabetes, inflammation and cardiovascular risk, and also exacerbates chronodisruption-related diseases, such as cancer and neurodegeneration (Melendez-Fernandez et al., 2023). In mouse models of neurodegenerative diseases, the light input into the circadian system often seems to be affected (Pfeffer et al., 2012, 2018; Weigel et al., 2023).

The central element of the circadian system is the circadian pacemaker, the suprachiasmatic nucleus (SCN) of the hypothalamus. It controls circadian rhythms via rhythmic signals and receives light information directly from the retina via the retinohypothalamic tract (RHT), which is responsible for photoentrainment (Johnson et al., 1988). The RHT is a monosynaptic pathway originating from intrinsically photo sensitive retinal ganglion cells (ipRGCs) that contain the photopigment melanopsin (Berson et al., 2002; Hattar et al., 2002). The ipRGCs play a key role in non-image-forming visual functions. This includes not only photoentrainment but also the pupillary light reflex and the modulation of mood (Fernandez et al., 2018). Although the ipRGCs co-release the neurotransmitters pituitary adenylate cyclase-activating peptide (PACAP) and glutamate, glutamate serves as the principal excitatory neurotransmitter mediating photoentrainment and negative masking responses to light (Purrier et al., 2014). PACAP modulates glutamatergic signaling within the SCN and contributes to light-induced activation of the immediate-early gene EGR1, which in turn has been implicated in regulating molecular clock function through BMAL1 oscillation (Riedel et al., 2020; Riedel et al., 2018). The RHT terminals form glutamatergic synapses mainly with SCN neurons located in the core region expressing the vasoactive-intestinal polypeptide (VIP) (Ebling, 1996). These synapses are enveloped by fine glial processes that significantly control the extracellular homeostasis of ions and glutamate, which is why it is also referred to as a “tripartite synapse” (Araque et al., 1999; Perea et al., 2009; Perez-Alvarez and Araque, 2013).

Glutamate transporters include the neuronal presynaptic vesicular glutamate transporters (VGLUT1 and VGLUT2), which are primarily localized to presynaptic terminals and mediate vesicular glutamate loading required for fast excitatory neurotransmission (Zhao et al., 2023). In contrast, the excitatory glutamate aspartate transporter (GLAST) is an integral plasma membrane protein predominantly expressed in astrocytes, where it contributes to glutamate uptake and homeostasis at tripartite synapses (Perego et al., 2000). Together, these transport systems are essential for efficient glutamatergic synaptic transmission in the adult mammalian brain. Both, VGLUT1 and VGLUT2 control synaptic strength by determining how much glutamate is loaded into synaptic vesicles, which directly influences quantal size, the amount of neurotransmitter released per vesicle fusion, (Herman et al., 2014; Wojcik et al., 2004) and also the release probability (Herman et al., 2014). However, they show a different distribution in the adult brain, different loading and release properties (Fremeau et al., 2004a,b; Herman et al., 2014). Vesicles with VGLUT2 seem to have a higher release probability than vesicles with VGLUT1 (Weston et al., 2011). VGLUT1 seems to be involved in the reserve pool and recycling of synaptic vesicles (Fremeau et al., 2004a,b). The quantitative analysis of VGLUT by confocal laser scanning microscopy allows a good approximation of the synaptic contacts (Melone et al., 2005). GLAST, indirectly regulates synaptic strength by rapidly clearing glutamate via fine astrocytic processes from the synaptic cleft after release, preventing spillover, desensitization of receptors, and excitotoxicity (Purushotham and Buskila, 2023). In vesicles prepared from the whole brain, VGLUT1 shows a circadian rhythm which seems to be controlled by the molecular clockwork (Yelamanchili et al., 2006). In SCN astrocytes, GLAST seems to modulate rhythmic neuronal activity (Brancaccio et al., 2017). A disrupted molecular clockwork in GLAST-positive astrocytes alters circadian locomotor activity and cognition (Barca-Mayo et al., 2017). Notably, disruptions in VGLUT1/2 and GLAST expression have been correlated with a range of neurological and psychiatric disorders, including schizophrenia, epilepsy, and neurodegenerative diseases (Ferrarese et al., 2000; Karlsson et al., 2009; Zhang et al., 2017), which are also associated with chronodisruption (Fishbein et al., 2021).

In the retinorecipient part of the SCN, VGLUT1 and VGLUT2 can be detected, with VGLUT2 being more strongly represented (Kiss et al., 2008). Ablation of VGLUT2 in ipRGCs results in strong impairment, but not complete elimination, of photoentrainment and negative masking, and in reduced stability of the circadian rhythm in locomotor activity in some mice (Purrier et al., 2014). However, with genetic ablation of VGLUT1, circadian photoentrainment is spared (Johnson et al., 2007). These findings suggest that VGLUT2 is more important for light input into the circadian system, although there appear to be redundancies. We could recently show that in the SCN of mice with a targeted deletion of the essential clock gene Bmal1, VGLUT1, VGLUT2 and GLAST are reduced while the distance of vesicles to the synaptic cleft in excitatory synapses and the glutamate concentration are increased, suggesting a role of the molecular clockwork in the homeostasis of glutamate released by the RHT tripartite synapse (Korkmaz et al., 2025). However, it is still not known whether glutamatergic transmission in the RHT synapse is modulated by the rhythmic environmental light conditions. Therefore, in this study, we investigated VGLUT1, VGLUT2 and GLAST using immunofluorescence and confocal laser microscopy in the SCN of mice kept under three different light paradigms: standard photoperiod, 12:12-h light–dark (LD), constant darkness (DD) and constant light (LL), in which we had already detected light-dependent changes in synaptic plasticity in the hippocampus (Schroder et al., 2023). Under LD, all three glutamate transporters showed a time of day-dependent rhythm, with a peak of VGLUT1 and GLAST in the light phase and a peak of VGLUT2 in the dark phase. This suggests that VGLUT1 and GLAST are more strongly coupled, whereas VGLUT2 may act as a complementary mechanism, so that the strength or efficiency of the light information can be modulated depending on the time of day. The rhythms of the neuronal glutamate transporters, but not the astrocytic glutamate transporter GLAST, were preserved under DD and LL, which suggests robust circadian control. Our study thus supports the hypothesis that the circadian system may regulate its own input. Understanding how glutamatergic signaling in the SCN adapts to changing light conditions is crucial for elucidating the mechanisms of chronodisruption.

## 2 Materials and methods

### 2.1 Experimental animals

Male C57Bl/6 J mice (8–12 weeks old) were obtained from Janvier Labs (Le Genest-Saint-Isle, France). Only male adult mice were included to minimize variability associated with the estrous cycle and to avoid developmental variation and effect of aging (Alvord et al., 2022; Goyette et al., 2023). Mice were housed in single standard cages in light- and sound-proof cabinets with automatic time switch (Beastmaster, Mannheim, Germany). Light exposure was set with an intensity of 400 lx during the light phase and <5 lx during the dark phase. All mice had free access to food and water *ad libitum*. The mice assignment to the three exposure groups was random. As the control group, Group 1 was exposed to a standard light cycle of 12 h light and 12 h darkness (LD) [light on at 6:00 a.m. = zeitgeber time (ZT) 00] for 16 days. Group 2 was maintained under LD cycle for 14 days and then placed in constant darkness (DD) [circadian time (CT) 00 = lights on in the former light phase] for at least 38 h before sacrifice. Group 3 was also kept under LD cycle for 14 days and then was exposed to constant light (LL) [disrupted time (DT) 12 = lights off in the former dark phase] for at least 38 h before being sacrificed. These short exposures to DD and LL serve to eliminate rhythmic light signals from the environment in order to unmask circadian rhythms without causing relevant phase shifts (Colwell, 2000). Mice from all groups were sacrificed every 4 h at six time points (02, 06, 10, 14, 18, and 22). Sampling started 2 h after lights on the LD group, 2 h after former lights on for the DD group and 2 h after former lights off for the LL group. Handling, anesthesia, and perfusion procedures were standardized across time points and lighting conditions in order to minimize variability. Each group consisted of 18 mice, 3 per time point. Each SCN (left and right) was analyzed, resulting in a total of 4–6 SCNs per each time point for statistical analysis, except for VGLUT at CT02/06 (2 SCNs). Every effort was made to minimize the number of animals used in accordance with the 3R principles. Mice were deeply anesthetized using ketamine/xylazine (100 mg/10 mg/kg body weight, respectively) and then transcardially perfused with 0.9% NaCl followed by 4% paraformaldehyde using a Ministar Peristaltic Pump (World Precision Instruments, Sarasota, FL, USA). Brains were removed from the skull, post-fixed for 24 h in 4% formalin. Brains were cut using a Vibratome (VT 1200S, Leica, Bensheim, Germany) in 50 μm thick free-floating sections.

All animal experiments were approved by the local government, North Rhine-Westphalia State Agency for Nature, Environment and Consumer Protection, Germany (Approval number: 84–02.04.2013. A358) and in agreement with the international guidelines on the ethical use of animals (Kilkenny et al., 2012). For this study, unstained coronal brain slices of the SCN region of animals, which we had already obtained in an earlier study (Schroder et al., 2023) were used, and thus, no new animals were sacrificed.

### 2.2 Immunofluorescence

Series of parallel coronal brain slices through the rostrocaudal extent of the SCN region were selected. One series from each of the three groups was stained for GLAST and one from each group was simultaneously stained for VGLUT1 and VGLUT2. Brain sections were incubated for 1 h with 5% normal goat serum (NGS) in PBS containing 0.2% Triton X-100, to reduce unspecific binding of the secondary antibody, and subsequently incubated for 48 h at 4 °C with rabbit anti-GLAST (1:1000, Cat. # ab416, Abcam, Cambridge, UK), guinea pig anti-VGLUT1 (1:2000, Cat. #135304, Synaptic System, Göttingen Germany), or rabbit anti-VGLUT2 (1:1000, Cat. #135402, Synaptic System, Göttingen, Germany) in PBS, 5% NGS, and 0.2% Triton X-100. After washing, sections were incubated for 1 h with Alexa 488-labeled goat anti-rabbit antibody (1:500, Invitrogen, Waltham, Massachusetts, USA), Alexa 488-labeled goat anti-guinea pig antibody (1:500, Invitrogen, Waltham, Massachusetts, USA), or Alexa 568-labeled goat anti-rabbit antibody (1:500, Invitrogen, Waltham, Massachusetts, USA) in PBS, 5% NGS, and 0.2% Triton X-100. DAPI (4′,6-Diamidino-2-phenylindol) was used to visualize cell nuclei (1:100 in PBS; 10 min). Sections were washed, transferred onto glass microscope slides, and covered with high precision glass coverslips using Fluoromount G (Southern Biotech, Birmingham, Alabama, USA). Information about the primary antibody validation are summarized in Table 1.

**Table 1:** List of primary antibody.

Primary antibody used	VGLUT1	VGLUT2	GLAST
Manufacturer	Synaptic systems	Synaptic systems	Abcam
Catalog number	135304	135402	ab416
RRID	AB_887878	AB_2187539	AB_304334
Immunogen	Recombinant protein corresponding to residues near the carboxy terminus of rat VGLUT 1	Synthetic peptide corresponding to residues near the carboxy terminus of rat VGLUT2	Synthetic Peptide within Rat Slc1a3. The exact immunogen used to generate this antibody is proprietary information.
Species reactivity	Rat, mouse, human, cow	Human, rat, mouse, chicken	Human, rat, mouse
Specificity	K.O. validated	–	–
Prior validation in mouse brain tissue	Souter et al. (2022), Eaker et al. (2026), Perdok et al. (2024)	Toledo et al. (2019), Aracri et al. (2013), Wang et al. (2025)	Huang et al. (2016), Skytt et al. (2016), Korkmaz et al. (2025)

### 2.3 Image acquisition and analysis

Image acquisition and analysis were performed by an investigator blinded to the experimental conditions. Confocal images were acquired using a Leica TCS SP8 laser-scanning microscope equipped with a Leica 63x glycerol-immersion objective lens (NA 1.3) (Leica, Bensheim, Germany) according to (Strehl et al., 2014). The pinhole was set to 1 Airy unit. All images (63x objective lens; 4x scan zoom) were acquired as single confocal optical sections and obtained at tissue levels ~ 5 μm below the surface. Microscope settings such as detector gain and amplifier offset were kept constant throughout image acquisition. For GLAST single labeling and VGLUT1/VGLUT2 double labeling, each confocal channel was analyzed independently to identify diffraction-limited immunoreactive puncta, representing localized fluorescence intensity maxima constrained by the optical resolution of the imaging system. In each SCN, six visual fields (each 1,024 × 1,024 pixels ≅ 46.18 μm x 46.18 μm = 2132.59 μm^2^) were analyzed. The theoretical lateral (XY) resolution of the system, corresponding to the point spread function, was ~190 nm for DAPI (405 nm excitation), ~230 nm for 488 nm excitation, and ~260 nm for 568 nm excitation under these imaging conditions.

The ImageJ software package for MacOS (version 1.53, downloaded on 24.10.2021 from https://imagej.net/software/fiji/) was used for quantitative analysis. A constant threshold value was set for each staining. All diffraction-limited immunopositive puncta with a minimum size of 0.04 μm^2^ and a circularity of 0.01–1.00 were defined as puncta and counted automatically. The intensity of immunofluorescence, the area and the density of the puncta in the six visual fields of the SCN were analyzed and averaged. The intensity of immunofluorescence was determined as arbitrary units (a.u.) by subtracting the mean gray value of the background from the mean gray value in the visual field. Puncta area was quantified as the apparent projected area of the diffraction-limited immunoreactive puncta and does not reflect true subcellular structural dimensions. The puncta density was expressed as the number of puncta per mm^2^ by dividing the number of puncta in the field of view by the area of the field of view.

### 2.4 Statistical analysis

24-h rhythm was evaluated by cosinor analysis using R 4.6.0 and RStudio 2,026.05.1 + 225 (R Foundation for Statistical Computing, Vienna, Austria). The left and right SCN was used as paired data obtained from one animal. Further statistical analysis was performed using GraphPad Prism software 8.3.1 (GraphPad Software, San Diego, California, USA). Prior to analysis, outliers were identified and excluded, and normality was assessed. As not all datasets were normally distributed, the non-parametric Kruskal–Wallis test followed by Dunn’s multiple comparisons were used to compare between the (former) light and (former) dark phases across different lighting conditions. For group comparisons, averaged value of the two SCNs were used and day and night time points were pooled. *p* values < 0.05 were considered statistically significant. Data are presented as mean ± standard error of the mean (SEM).

## 3 Result

### 3.1 VGLUT1 immunofluorescence intensity, puncta area and density

**Figure 1:**
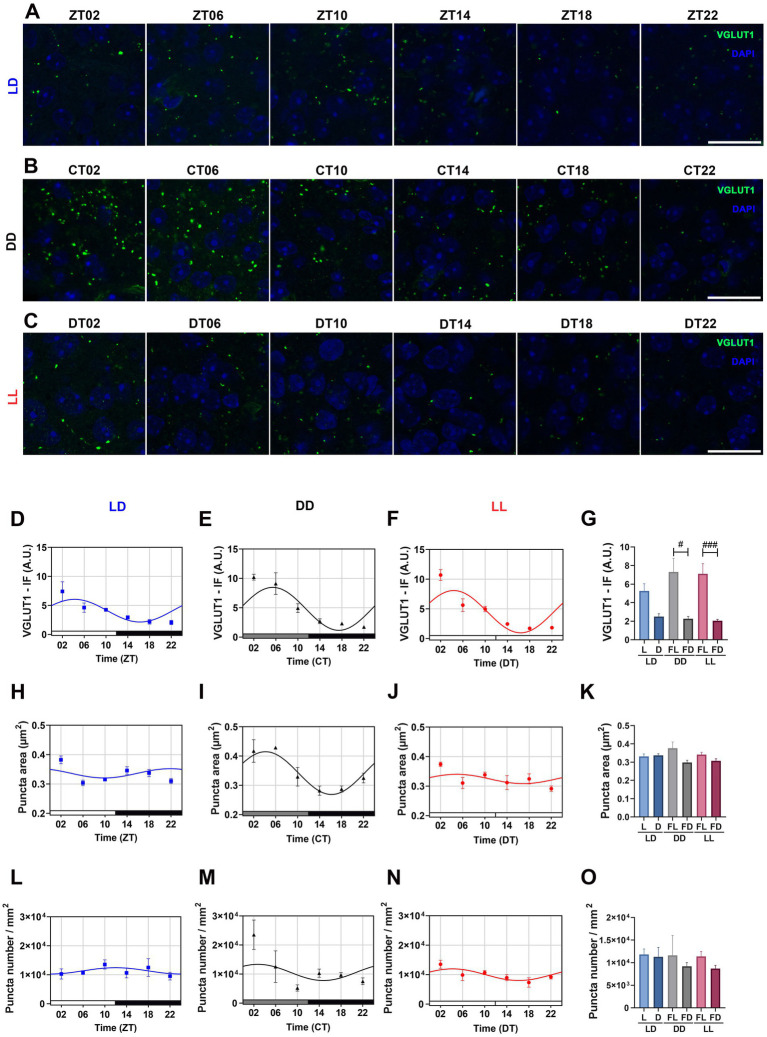
Twenty-four-hour cosinor analysis of VGLUT1-immunofluorescence intensity, puncta area, and puncta density in the suprachiasmatic nucleus (SCN). Representative confocal laser microscopic images of VGLUT1- immunofluorescence (IF) (green) and DAPI-stained nuclei (blue) in the SCN of mice kept **(A)** in a standard photoperiod (LD) of 12 h light and 12 h darkness and killed at 6 different zeitgeber times (ZT); **(B)** in constant darkness (DD) and killed at 6 different circadian times (CT); **(C)** in constant light (LL) and killed at 6 different disturbed times (DT). Scale bar, 20 μm. Quantitative analysis of VGLUT1 immunofluorescence intensity **(D–F)**, puncta area **(H–J)**, and puncta density **(L–N)** using cosinor fit with a fixed 24-h period (solid line). Symbols represent the mean ± SEM values. Comparison of **(G)** VGLUT1-IF, **(K)** VGLUT1 puncta area, **(O)** and VGLUT1 puncta density in the SCN of mice kept in LD and killed in the light (L) or dark (D) phase (pooled 3 time points each) and of mice kept in DD, and LL killed in the former light (FL) and former dark (FD) phase (pooled 3 time points each). Symbols and bars represent the mean ± SEM. Dunn’s multiple comparison, difference between pooled time points ^#^*p* < 0.05, ^###^*p* < 0.001.

The *intensity* of VGLUT1 immunofluorescence (Figures 1A–C) exhibited significant 24-h rhythmicity under LD (*p* = 0.001), DD (*p* = <0.0001), and under LL (*p* = <0.0001) conditions as determined by cosinor analysis (Figures 1D–F). The estimated acrophase occurred during the light phase under LD (ZT 04.3) (Figure 1D) and during the corresponding former light phase under DD (CT 05.5) (Figure 1E) and LL (DT 04.4) (Figure 1F). When the respective time points were pooled, the intensity of VGLUT1 immunofluorescence was higher in the (former) light phase than in the (former) dark phase of the DD and LL group but not significantly different among the groups (Figure 1G).

The *area* of VGLUT1 puncta exhibited significant 24-h rhythmicity under and DD (*p* = <0.0001), but not under LD (*p* = 0.267) and LL (*p* = 0.354) conditions as determined by cosinor analysis (Figures 1H–J). The estimated acrophase occurred during the former light phase under DD (CT 04.2) (Figure 1I). When the respective time points were pooled, the VGLUT1 puncta area did not differ significantly between the (former) light and (former) dark phases or among the three lighting conditions (Figure 1K).

In contrast, the *density* of VGLUT1 puncta did not exhibited significant 24-h rhythmicity under LD (*p* = 0.7083), DD (*p* = 0.256), or LL (*p* = 0.056) conditions as determined by cosinor analysis (Figures 1L–N). When the respective time points were combined, the density of the VGLUT1 puncta did not differ between the (former) light phase and the (former) dark phase, nor among the groups (Figure 1O).

### 3.2 VGLUT2 immunofluorescence intensity, puncta area and density

**Figure 2:**
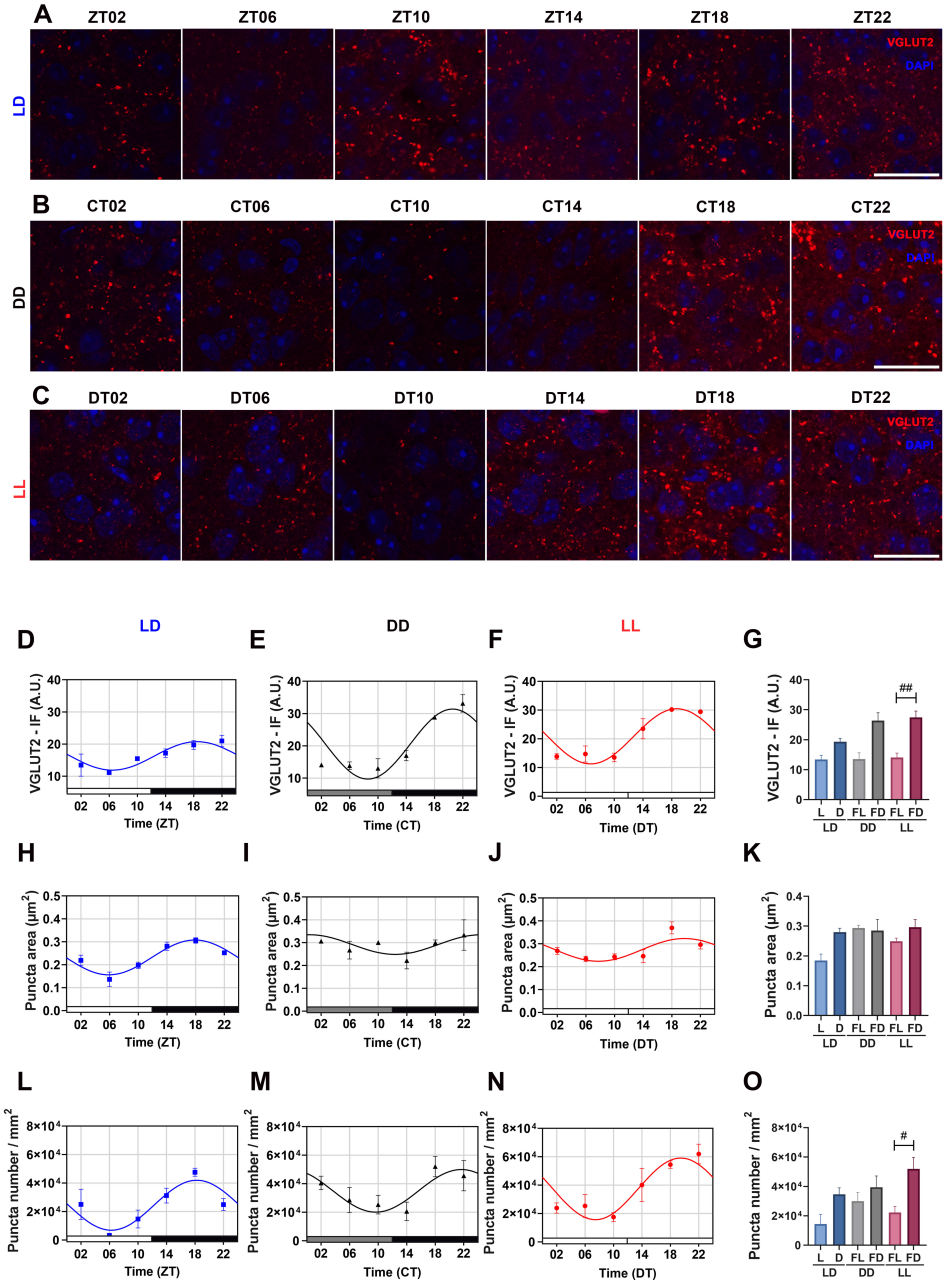
Twenty-four-hour cosinor analysis of VGLUT2-immunofluorescence intensity, puncta area, and puncta density in the suprachiasmatic nucleus (SCN). Representative confocal laser microscopic images of VGLUT2- immunofluorescence (IF) (green) and DAPI-stained nuclei (blue) in the SCN of mice kept **(A)** in a standard photoperiod (LD) of 12 h light and 12 h darkness and killed at 6 different zeitgeber times (ZT); **(B)** in constant darkness (DD) and killed at 6 different circadian times (CT); **(C)** in constant light (LL) and killed at 6 different disturbed times (DT). Scale bar, 20 μm. Quantitative analysis of VGLUT2 immunofluorescence intensity **(D–F)**, puncta area **(H–J)**, and puncta density **(L–N)** using cosinor fit with a fixed 24-h period (solid line). Symbols represent the mean ± SEM values. Comparison of **(G)** VGLUT2-IF, **(K)** VGLUT2 puncta area, **(O)** and VGLUT2 puncta density in the SCN of mice kept in LD and killed in the light (L) or dark (D) phase (pooled 3 time points each) and of mice kept in DD, and LL killed in the former light (FL) and former dark (FD) phase (pooled 3 time points each). Symbols and bars represent the mean ± SEM. Dunn’s multiple comparison, difference between pooled time points ^#^*p* < 0.05, ^##^*p* < 0.01.

The *intensity* of VGLUT2 immunofluorescence (Figures 2A–C) exhibited significant 24-h rhythmicity under LD (*p* = 0.001), DD (*p* = <0.0001), and under LL (*p* = <0.0001) conditions as determined by cosinor analysis (Figures 2D–F). The estimated acrophase occurred during the dark phase of LD (ZT 18.4) (Figure 2D) and during the corresponding former dark phase under DD (CT 20.6) (Figure 2E) and LL (DT 18.7) (Figure 2F). When the respective time points were pooled, the intensity of VGLUT2 immunofluorescence was lower in the former light phase than in the former dark phase of the LL group but not significantly different among the groups (Figure 2G).

The *area* of VGLUT2 puncta exhibited significant 24-h rhythmicity under LD (*p* = <0.0001) and LL (*p* = 0.002), but not under DD (*p* = 0.265) conditions as determined by cosinor analysis (Figures 2H–J). The estimated acrophase occurred during the dark phase under LD (ZT 17.9) (Figure 2H) and during the subjective dark phase under LL (DT 19.8) (Figure 2J). When the respective time points were pooled, the VGLUT2 puncta area did not differ significantly between the (former) light and (former) dark phases or among the three lighting conditions (Figure 2K).

The *density* of VGLUT2 puncta exhibited significant 24-h rhythmicity under LD (*p* = <0.001), DD (*p* = 0.024), and under LL (*p* = <0.001) conditions as determined by cosinor analysis (Figures 2L–N). The estimated acrophase occurred during the dark phase under LD (ZT 18.2) (Figure 2L) and during the corresponding subjective dark phase under DD (CT 21.6) (Figure 2M) and LL (DT 19.5) (Figure 2N). When the respective time points were pooled, the density of the VGLUT2 puncta was lower in the former light phase compared to the former dark phase of the LL group but not significantly different among the groups (Figure 2O).

### 3.3 GLAST immunofluorescence intensity, puncta area and density

**Figure 3:**
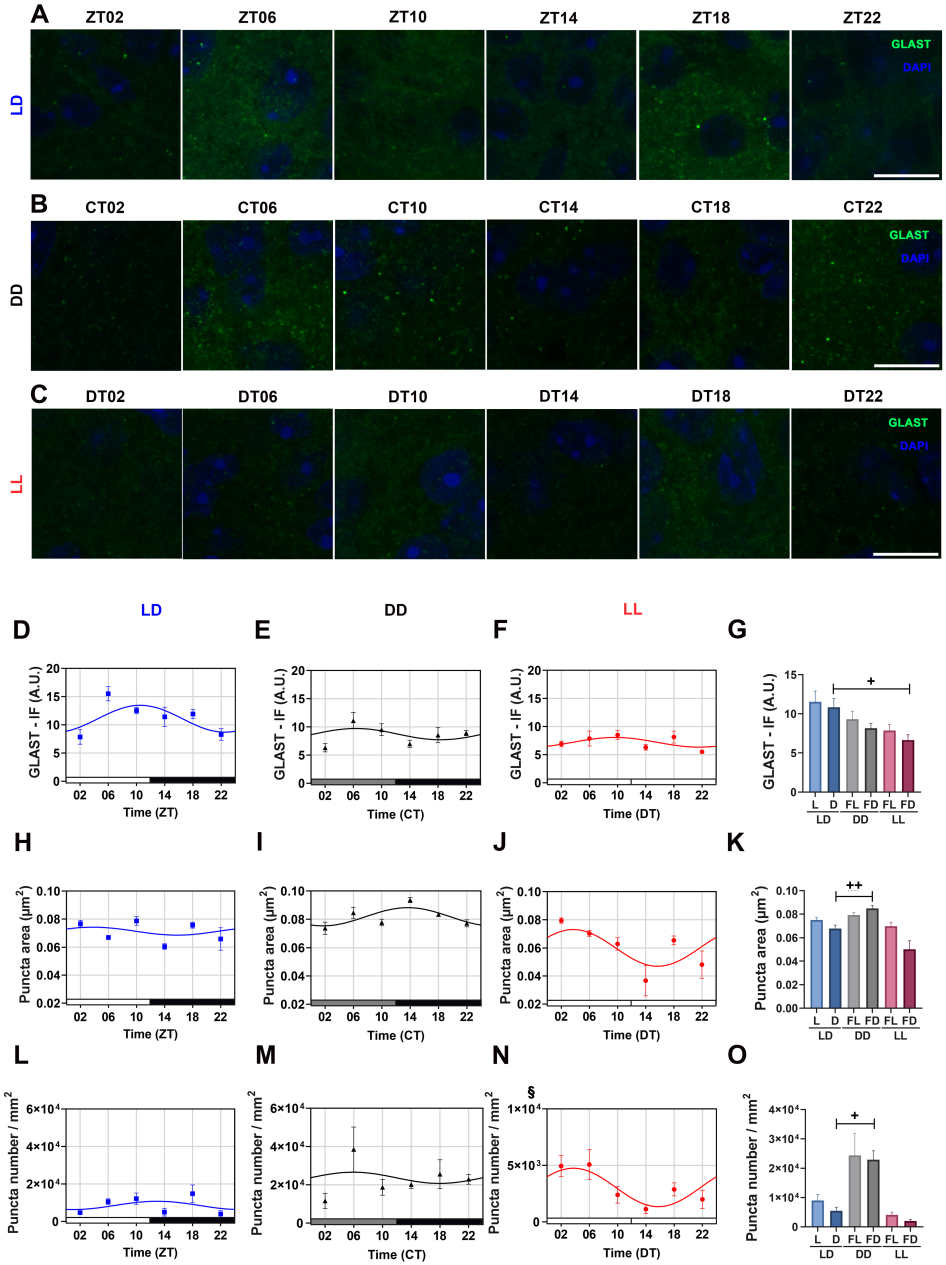
Twenty-four-hour cosinor analysis of GLAST-immunofluorescence intensity, puncta area, and puncta density in the suprachiasmatic nucleus (SCN). Representative confocal laser microscopic images of GLAST- immunofluorescence (IF) (green) and DAPI-stained nuclei (blue) in the SCN of mice kept **(A)** in a standard photoperiod (LD) of 12 h light and 12 h darkness and killed at 6 different zeitgeber times (ZT); **(B)** in constant darkness (DD) and killed at 6 different circadian times (CT); **(C)** in constant light (LL) and killed at 6 different disturbed times (DT). Scale bar, 20 μm. Quantitative analysis of GLAST immunofluorescence intensity **(D–F)**, puncta area **(H–J)**, and puncta density **(L–N)** using cosinor fit with a fixed 24-h period (solid line). Symbols represent the mean ± SEM values. Comparison of **(G)** GLAST-IF, **(K)** GLAST puncta area, **(O)** and GLAST puncta density in the SCN of mice kept in LD and killed in the light (L) or dark (D) phase (pooled 3 time points each) and of mice kept in DD, and LL killed in the former light (FL) and former dark (FD) phase (pooled 3 time points each). Symbols and bars represent the mean ± SEM. Dunn’s multiple comparison, differences between groups ^+^*p* < 0.05, ^++^*p* < 0.01. ^§^Note the different scale.

The *intensity* of GLAST immunofluorescence (Figures 3A–C) exhibited significant 24-h rhythmicity under LD (*p* = 0.023) but not under DD (*p* = 0.331), or LL (*p* = 0.272) conditions as determined by cosinor analysis (Figures 3D–F). The estimated acrophase occurred during the light phase under LD (ZT 10.5) (Figure 3D). When the respective time points were pooled, the intensity of GLAST immunofluorescence was not different between the (former) light and (former) dark phase of the LD, DD and LL group. However, it was significantly higher in the dark phase of the LD group than in the former dark phase of the LL group (Figure 3G).

The *area* of GLAST puncta exhibited significant 24-h rhythmicity under DD (*p* = 0.011), and LL (*p* = 0.032) but not under LD (*p* = 0.524) conditions as determined by cosinor analysis (Figures 3H–J). The estimated acrophase occurred during the former dark phase under DD (CT 13.7) (Figure 3I) and during the former light phase under LL (DT 03.7) (Figure 3J). When the respective time points were pooled, the GLAST puncta area did not show significant differences between the (former) light and (former) dark phases of all groups. (Figure 3K). However, the GLAST puncta were larger in the former dark phase of the DD group than in the dark phase of the LD group (Figure 3K).

The *density* of GLAST puncta exhibited significant 24-h rhythmicity under LL (*p* = 0.01), but not under LD (*p* = 0.491) or DD (*p* = 0.77) conditions as determined by cosinor analysis (Figures 3L–N). The estimated acrophase occurred during the former light phase under LL (DT 03.7) (Figure 3N). When the respective time points were pooled, the density of the GLAST puncta was not significant between the (former) light and (former) dark phase, however it was significantly lower in the dark phase of the LD group than in the former dark phase of the DD group (Figure 3O).

## 4 Discussion

Glutamatergic signal transmission from the retina to the SCN is essential for the photoentrainment of diverse circadian rhythms. Glutamate transporters in the presynapse (VGLUT1 and 2) but also in the astrocyte end feet (GLAST), which envelop the synapses, are key structures of glutamatergic signal transmission. We see 24-h rhythm of VGLUT1, VGLUT2 and GLAST in the SCN of the mouse. VGLUT1 and GLAST show a peak in the light phase, while VGLUT2 shows a peak in the dark phase. This suggests that VGLUT1 and GLAST are temporally coupled and that VGLUT2 is a complementary system. The rhythms of neuronal glutamate transporters are conserved under DD and LL, suggesting robust circadian control. In contrast, the rhythm of the astrocytic GLAST was not conserved under DD and LL. Similar to the SCN of the rat (Kiss et al., 2008), the expression of VGLUT1 was significantly lower than that of VGLUT2 in the SCN of the mouse. Although VGLUT1 and VGLUT2 have a different distribution pattern in the adult brain (Balaram et al., 2013; Fremeau et al., 2004a,b), both VGLUTs can also occur in the same neuron (De Gois et al., 2005), in the same presynapse, and also in the same vesicle pool (Herzog et al., 2006).

The expression level of VGLUT1 determines the amount of glutamate that is loaded into the vesicles and then released into the synaptic cleft, thereby regulating the effectiveness of neurotransmission (Wojcik et al., 2004). Therefore, we can assume that the upregulation of VGLUT1 in the light phase reflects the upregulation of the quantal size. Our findings are in line with the fact that the transmission of light information from the ipRGCs to the SCN occurs predominantly by glutamate (Ebling, 1996). The light information of the ipRGCs plays an important role in photoentrainment, for which nocturnal rodents actively collect the light information at the beginning of the light phase (Steel et al., 2024). On the other hand, it leads to an inhibition of activity and sleep induction (Muindi et al., 2014). Synaptic vesicles obtained from the entire mouse brain also show a time-of-day fluctuation of VGLUT1 with a peak shortly before the start of the light phase (Yelamanchili et al., 2006). In addition, synaptic vesicles obtained from the entire mouse brain show diurnal rhythms in the sorting of VGLUT1 at the plasma membrane (Darna et al., 2009) and VGLUT1-mediated endocytosis, in which interaction with endophilin and phosphorylation of dynamin play a phase-dependent role (Richter et al., 2018). The study by Richter et al. (2018) suggests that the synaptic vesicles throughout the mouse brain are efficiently charged with glutamate in the light/inactive phase, but have a low release probability due to a higher endophilin-mediated endocytosis, while there are fewer vesicles in the release-capable pool in the dark/active phase. Synaptopodin, which is essential for the formation of the spine apparatus in dendritic spines (Vlachos et al., 2013), shows a higher density in the light phase in the hippocampus of mice compared to the dark phase (Schroder et al., 2023), indicating a light-dependent modulation of the synaptic plasticity of hippocampal excitatory neurons. Since the rhythmic changes in the environment require anticipation and/or adaptation of neurotransmission (Albrecht and Eichele, 2003; Tsanov and Manahan-Vaughan, 2007), it can be assumed that the dynamics of the glutamatergic vesicles in the entire brain are related to the anticipation or perception of the light phase. Glutamate transmits direct or indirect light information mediated by the SCN from the ipRGCs (LeGates et al., 2014) but also from subcortical visual core areas and from the visual cortex. Glutamate is the essential excitatory transmitter in the brain and, accordingly, also plays an essential role in other functional systems, including the motor system, which are subject to circadian rhythms.

In the SCN of mice kept under acute constant darkness, both VGLUT1-IF intensity and puncta area exhibited significant circadian rhythmicity, with acrophases remaining in the subjective light phase. Importantly, the VGLUT1 puncta represent a correlate for the presynapse (Melone et al., 2005), this could indicate a temporally dynamic regulation of synapse size. Also, in the SCN of mice kept under acute constant light, VGLUT1-IF intensity retained a significant 24-h rhythm peaking in the former light phase, indicating preservation of its temporal phase despite continuous light exposure. In contrast, puncta area and density do not exhibit significant rhythmicity. This indicates that even without a rhythm in the ambient lighting, rather in anticipation of the light phase, there is an upregulation of VGLUT1 expression in the individual synapses in the SCN. The regulation of VGLUT1-positive synapses in the SCN therefore seems to be under endogenous circadian control. This is in line with our recent study, which showed that in the SCN of mice with a defective molecular clockwork (Bmal1−/−), VGLUT1 expression is reduced, which is associated with a decrease in the readily releasable pool in excitatory SCN synapses and altered light-dependent behavior (Korkmaz et al., 2025). In synaptic vesicles from the entire brain of mice with a defective molecular clockwork (Per2^Brdm1^), the diurnal rhythms of VGLUT1 expression (Yelamanchili et al., 2006), the sorting of VGLUT1 at the plasma membrane (Darna et al., 2009) and VGLUT1-mediated endocytosis (Richter et al., 2018) are disturbed. However, we do not see a rhythm in the expression of the postsynaptic protein synaptopodin in the hippocampus of DD mice (Schroder et al., 2023). These findings indicate that the molecular clockwork is involved in rhythmic glutamatergic neurotransmission at the presynaptic level not only in the SCN but also in other brain regions. Consistently, under LD conditions, the light phase predominance of VGLUT1 and GLAST is broadly aligned with rhythms of Per1 and Per2 mRNA and protein expression in the SCN, while VGLUT2 is peaking during the same circadian phase of Bmal1 mRNA expression during the night (Honma, 2018). Together with our previous observation that VGLUT1, VGLUT2, and GLAST expression are reduced in Bmal1-deficient mice (Korkmaz et al., 2025), these findings support a potential relationship between the molecular clockwork and glutamatergic transporter regulation. Neuroplasticity of the neocortex exhibits circadian rhythmicity, which is reflected by rhythmic plasticity markers controlled by a combination of the central clock of the SCN and a local cortical circadian oscillator (Bille et al., 2025). Bmal1 is localized in both the pre- and postsynapse of hippocampal neurons and appears to interact primarily with calmodulin kinase (CaMKIIa) (Barone et al., 2023), which are associated with synaptic organization, function, and plasticity, learning and memory, sleep, and circadian rhythms (Barone et al., 2023) as well as in the regulation of VGLUT1 expression (Melo et al., 2013).

The intensity of VGLUT2 immunofluorescence as well as the area and density of VGLUT2-positive puncta in the SCN of mice maintained under a 12-h light/12-h dark cycle exhibited significant diurnal rhythmicity, peaking during the dark phase, thus in antiphasic relation to VGLUT1. VGLUT2-expressing vesicles show a high probability of release (Fremeau et al., 2001, 2004a,b; Weston et al., 2011) In contrast, VGLUT1 has been shown to interact with endocytic proteins such as endophilin, suggesting differences in vesicle trafficking pathways (De Gois et al., 2006; Vinatier et al., 2006; Voglmaier and Edwards, 2007). These molecular differences raise the possibility that time-of-day–dependent changes in quantal size or vesicle dynamics may occur, although this remains to be directly tested. As with VGLUT1, the VGLUT2 rhythm was maintained in DD and LL, indicating that it is also controlled endogenously, independent of the rhythmic ambient lighting. This is consistent with VGLUT2 expression that was also reduced in the SCN of Bmal1−/− mice (Korkmaz et al., 2025), further supporting regulation by the molecular clock. VGLUT1 and VGLUT2 show bidirectional and opposite regulation in response to neuronal activity (De Gois et al., 2005). In this context, it is notable that the inner retina displays circadian rhythms in electrical activity (Storch et al., 2007). This raises the possibility that rhythmic retinal output may contribute to the antiphasic expression patterns of VGLUT1 and VGLUT2 observed in the SCN. Since this retinal activity is itself under circadian control, it may represent one upstream factor influencing rhythmic presynaptic VGLUT expression in SCN afferents. However, the precise mechanisms linking retinal activity, circadian clock function, and VGLUT regulation remain to be established.

The prominent role of VGLUT2 in retinal ganglion cell projections to the SCN, together with evidence that its loss disrupts photic entrainment, suggests that VGLUT2 mediates the primary excitatory light input to the circadian system. Furthermore, recent work mapped FOS- and EGR1-positive SCN neurons using an early-night light pulse and showed that photic activation is concentrated in SCN core neuron populations, including a subset of neurons in the shell (Riedel et al., 2023). In this context, the dark-phase predominance of VGLUT2 we observed aligns temporally with the phase at which photic stimulation most effectively recruits these postsynaptic SCN populations. This supports the idea that circadian regulation of VGLUT2 contributes to time-of-day-dependent tuning of the efficacy of retinal light input to the SCN. Although the anatomical distribution of vesicular glutamate transporters is largely preserved (Vigneault et al., 2015), the conservation of their circadian regulation among nocturnal and diurnal species, and thus their role in sleep and behavior, remains unclear. The presence of rhythmic VGLUT1 expression in the SCN, despite its apparent dispensability for photoentrainment, raises the question of its functional significance. One possibility is that VGLUT1 contributes to the modulation of synaptic gain or intra-SCN network communication, whereas VGLUT2 likely mediates the primary excitatory retinal input. Functional redundancy and differential release probabilities may further ensure robustness of light entrainment. These interpretations remain hypothetical and will require functional studies to determine the specific contributions of each transporter.

Efficient glutamatergic transmission in the SCN depends not only on the proper functioning of neuronal vesicular glutamate transporters (VGLUT1 and VGLUT2), but also on astrocytic glutamate transporters such as GLAST. Glutamate released by the ipRCGS in the SCN is eliminated by the astrocytes by means of transporters including GLAST, which limits spillover, preserves signal fidelity, and prevents excitotoxicity. In astrocytes, glutamate is converted to glutamine by means of glutamine synthetase, which is released back to neurons. Under LD conditions, GLAST-IF in the SCN exhibited significant diurnal rhythmicity, peaking during the middle of the light phase and thereby sharing the same temporal phase as VGLUT1. This is consistent with *Glast* mRNA and GLAST protein (Spanagel et al., 2005) as well as glutamate uptake and glutamine synthetase activity (Leone et al., 2015) are significantly higher in the light phase than in the dark phase in the mouse SCN. The diurnal variations of GLAST-IF were not maintained under DD and LL, indicating that it may be regulated by light/dark cycles rather than endogenously controlled. In line with this, *Glast* mRNA expression does not seem to be directly mediated by E-box elements (Spanagel et al., 2005). Interestingly, in the mouse SCN, the higher activity of astrocytic GLAST and EAAT2 during the day seems to increase neuronal activity (Brancaccio et al., 2017) and the astrocytic molecular clockwork might even be sufficient to control circadian rhythms of SCN neurons and in behavior via glutamatergic signals (Brancaccio et al., 2019). Importantly, the kinetics of glutamatergic neurotransmission is shaped by multiple factors including not only glutamate release and astrocytic clearance, but also diffusion rates, receptor binding as well as unbinding rate, and desensitization that are regulated on the millisecond timescale (Cuellar-Santoyo et al., 2022; Robert and Howe, 2003; Rusakov and Stewart, 2021; Traynelis et al., 2010).

Although in the present study does not assess a direct circadian readout, e.g., long-term free-running activity monitoring or sleep–wake cycles via EEG/EMG recordings, our previous work demonstrated persistent circadian rhythms of spontaneous locomotor activity and plasma corticosterone levels in the same animal cohorts (Schroder et al., 2023) under LD, DD, and LL conditions. Thus, the observed rhythms in glutamatergic transporters occurred in the context of an intact circadian system. Importantly, the LL mice showed increased cortisol levels as well as reduced cognitive performance and hippocampal plasticity (Schroder et al., 2023). In this study, we show decreased GLAST-IF in the SCN of LL mice. These findings are particularly relevant in the context of modern society, where exposure to non-natural light cycles such as shift work and ALAN is becoming more common, which is associated with sleep disturbances and an increased risk of chronic disease (Korf et al., 2025). Further studies are necessery to show whether continuous light also affects neuronal and glial glutamatergic transmission in humans, especially in the non-image forming visual system, which seems to be particularly important for mood regulation (LeGates et al., 2014).

### 4.1 Limitations and outlook

In this study, we used C57BL/6 J mice, as this is the most widely used mouse strain, particularly for the generation of transgenic mice. However, we would like to note that this strain—like most inbred mouse strains—are melatonin deficient. Although this deficiency does not significantly affect activity rhythms or the molecular clockwork within the SCN (Korf and von Gall, 2024), a comparative analysis with a melatonin-proficient mouse strain would be of interest for future research. Another limitation of our study was the small sample size. However, in accordance with strict animal welfare regulations (3Rs: Replacement, Reduction, and Refinement), we had to minimize the number of animals used, particularly for time-series analyses. To compensate for this, every possible measure was taken to reduce variability. This also resulted in a further limitation: the use of only male mice, which, unlike female mice, do not exhibit significant fluctuations of sex hormones that could influence circadian rhythms and glutamatergic neurotransmission (Alvord et al., 2022; Goyette et al., 2023). Future studies incorporating female animals will be important to evaluate sex-dependent effects and interactions between hormonal state and circadian synaptic regulation. Furthermore, this study primarily relies on correlative immunofluorescence data to assess circadian changes in glutamate transporter expression in the SCN. While these findings are consistent with a potential role in modulating photic input, direct functional and causal evidence was not addressed here and will require future investigation using electrophysiological, behavioral, and targeted manipulation approaches. The persistence of rhythmicity of VGLUT1 and VGLUT2 immunofluorescence under constant conditions supports endogenous circadian regulation, although its precise functional impact on light-input efficacy remains to be determined.

## 5 Conclusion

In conclusion, the neuronal vesicular glutamate transporters VGLUT1 and VGLUT2 as well as the astrocytic glutamate transporter GLAST show 24-h rhythms in the SCN of the mouse, in which GLAST and VGLUT1 have an opposite phase position to VGLUT2. In addition, the neuronal glutamate transporter maintained rhythmic changes under constant light conditions. Thus, there is circadian control of rhythmic plasticity of glutamatergic transmission in the SCN, which might serve on the one hand for the photoentrainment and on the other hand for the timekeeping mechanism. Continuous light has a suppressive effect on GLAST, which could be important in the context of an altered glutamatergic transmission in the non-image forming visual system by ALAN in humans.

## Data Availability

The datasets presented in this study can be found in online repositories. The names of the repository/repositories and accession number(s) can be found at: https://researchdata.hhu.de/handle/entry/216.
